# The influence of autistic symptoms on social and non-social cognition and on real-life functioning in people with schizophrenia: Evidence from the Italian Network for Research on Psychoses multicenter study

**DOI:** 10.1192/j.eurpsy.2020.99

**Published:** 2020-11-10

**Authors:** Antonio Vita, Stefano Barlati, Giacomo Deste, Paola Rocca, Alessandro Rossi, Alessandro Bertolino, Eugenio Aguglia, Mario Amore, Antonello Bellomo, Massimo Biondi, Bernardo Carpiniello, Enrico Collantoni, Alessandro Cuomo, Enrico D’Ambrosio, Liliana dell’ Osso, Massimo di Giannantonio, Giulia Maria Giordano, Carlo Marchesi, Palmiero Monteleone, Cristiana Montemagni, Lucio Oldani, Maurizio Pompili, Rita Roncone, Rodolfo Rossi, Alberto Siracusano, Patrizia Zeppegno, Gabriele Nibbio, Silvana Galderisi, Mario Maj, Italian Network for Research on Psychoses

**Affiliations:** 1 Department of Clinical and Experimental Sciences, University of Brescia, Brescia, Italy; 2 Department of Mental Health and Addiction Services, ASST-Spedali Civili, Brescia, Italy; 3 Department of Neuroscience, Section of Psychiatry, University of Turin, Turin, Italy; 4 Section of Psychiatry, Department of Biotechnological and Applied Clinical Sciences, University of L’Aquila, L’Aquila, Italy; 5 Department of Neurological and Psychiatric Sciences, University of Bari, Bari, Italy; 6 Department of Clinical and Molecular Biomedicine, Psychiatry Unit, University of Catania, Catania, Italy; 7 Section of Psychiatry, Department of Neurosciences, Rehabilitation, Ophthalmology, Genetics and Maternal and Child Health, University of Genoa, Genoa, Italy; 8 Psychiatry Unit, Department of Medical Sciences, University of Foggia, Foggia, Italy; 9 Department of Neurology and Psychiatry, Sapienza University of Rome, Rome, Italy; 10 Section of Psychiatry, Department of Public Health, Clinical and Molecular Medicine, University of Cagliari, Cagliari, Italy; 11 Psychiatric Clinic, Department of Neurosciences, University of Padua, Padua, Italy; 12 Department of Molecular Medicine and Clinical Department of Mental Health, University of Siena, Siena, Italy; 13 Section of Psychiatry, Department of Clinical and Experimental Medicine, University of Pisa, Pisa, Italy; 14 Department of Neuroscience and Imaging, G. D’Annunzio University, Chieti, Italy; 15 Department of Psychiatry, University of Campania “Luigi Vanvitelli”, Naples, Italy; 16 Department of Neuroscience, Psychiatry Unit, University of Parma, Parma, Italy; 17 Department of Medicine, Surgery and Dentistry “Scuola Medica Salernitana”, Section of Neuroscience, University of Salerno, Salerno, Italy; 18 Department of Psychiatry, University of Milan, Milan, Italy; 19 Department of Neurosciences, Mental Health and Sensory Organs, S. Andrea Hospital, Sapienza University of Rome, Rome, Italy; 20 Unit of Psychiatry, Department of Life, Health and Environmental Sciences, University of L’Aquila, L’Aquila, Italy; 21 Department of Systems Medicine, Psychiatry and Clinical Psychology Unit, Tor Vergata University of Rome, Rome, Italy; 22 Department of Translational Medicine, Psychiatric Unit, University of Eastern Piedmont, Novara, Italy

**Keywords:** autism spectrum disorders, cognition, psychosocial functioning, schizophrenia, social cognition

## Abstract

**Background:**

Autism spectrum disorders (ASDs) and schizophrenia spectrum disorders (SSDs), although conceptualized as separate entities, may share some clinical and neurobiological features. ASD symptoms may have a relevant role in determining a more severe clinical presentation of schizophrenic disorder but their relationships with cognitive aspects and functional outcomes of the disease remain to be addressed in large samples of individuals.

**Aims:**

To investigate the clinical, cognitive, and functional correlates of ASD symptoms in a large sample of people diagnosed with schizophrenia.

**Methods:**

The severity of ASD symptoms was measured with the PANSS Autism Severity Scale (PAUSS) in 921 individuals recruited for the Italian Network for Research on Psychoses multicenter study. Based on the PAUSS scores, three groups of subjects were compared on a wide array of cognitive and functional measures.

**Results:**

Subjects with more severe ASD symptoms showed a poorer performance in the processing speed (*p* = 0.010), attention (*p* = 0.011), verbal memory (*p* = 0.035), and social cognition (*p* = 0.001) domains, and an overall lower global cognitive composite score (*p* = 0.010). Subjects with more severe ASD symptoms also showed poorer functional capacity (*p* = 0.004), real-world interpersonal relationships (*p* < 0.001), and participation in community-living activities (*p* < 0.001).

**Conclusions:**

These findings strengthen the notion that ASD symptoms may have a relevant impact on different aspects of the disease, crucial to the life of people with schizophrenia. Prominent ASD symptoms may characterize a specific subpopulation of individuals with SSD.

## Introduction

### Background

Autism spectrum disorders (ASDs) and schizophrenia spectrum disorders (SSDs) are currently conceptualized as separate nosological entities [1]. However, this dichotomic separation has been called into question, as the two spectra show many similarities, and their overlap has recently been the focus of a growing body of literature [2–7]. In fact, one of the earliest conceptualizations of schizophrenia, redacted by Eugen Bleuler over a century ago [8], already described autistic features as a central element of the disorder, and only later was ASD defined as a distinct entity.

Deficits in social cognition and social interactions are key features of both ASDs and SSDs [9,10], and different brain imaging and genetic studies suggest that the two spectra might share similar aspects not only at a clinical level but also at a neurobiological, pathophysiological, and etiopathogenetic levels [11–15].

ASDs symptoms are more frequent in people diagnosed with schizophrenia than in healthy subjects [16,17], and, in people with SSDs, more severe ASD symptoms emerged as predictors of poorer performance on different measures of social cognitive abilities, both in the emotion processing and in the mental state attribution/theory of mind domains [18]. Prominent ASD symptoms have also been linked to poorer real-world functioning and greater impairments in the ability to judge the quality of everyday functioning [19].

Furthermore, a recent study investigating cognitive and clinical correlates of ASD symptoms in schizophrenia has found that people with a clear diagnosis of schizophrenia and prominent ASD symptoms showed a lower IQ and a poorer performance in a number of cognitive domains, including processing speed, working memory, and executive functions, leading to the interesting hypothesis that these subjects diagnosed with schizophrenia may represent a subpopulation, with specific clinical characteristics [20]. In addition, another study demonstrated a poorer response to antipsychotic treatment in first-episode early-onset psychosis patients with ASD, compared to those without ASD [21].

Thus, the investigation of the presence of ASD symptoms in people diagnosed with schizophrenia represents an interesting and important issue for the study of the illness itself and for the development of more tailored interventions. However, the most used diagnostic instruments available for the assessment of ASD, namely the autism diagnostic observation schedule (ADOS) [22] and the autism diagnostic interview-revised (ADI-R) [23], may not represent a viable solution for the assessment of ASD symptoms in people with schizophrenia, due to the complexity and time required for their application. Recently, the PANSS Autism Severity Score (PAUSS) [24], a scale derived from the Positive and Negative Syndrome Scale (PANSS) [25], has been developed and demonstrated to be an easy and reliable instrument for the assessment of ASD symptoms in people diagnosed with schizophrenia in the clinical practice.

A recent study [26] confirmed, in a small sample of patients recruited for a cognitive remediation study, that the PAUSS represents a valid and practical instrument for the assessment of ASD symptoms in people diagnosed with schizophrenia, comparable to more established but more complex and time-consuming tools as the ADOS and the ADI-R. Moreover, using the PAUSS cut-off score for “autistic schizophrenia” [24], it was possible to identify a subgroup of patients with schizophrenia and ASD symptoms, characterized by a lower IQ, poorer neuro- and socio-cognitive performance, and poorer real-world functioning. Although being of both theoretical and clinical interest, these findings have to be replicated in larger samples, better representing the population of people diagnosed with schizophrenia.

### Aims

The aim of the present study was to further investigate the clinical, cognitive, and functional correlates of ASD symptoms, as assessed with the PAUSS, in a large sample of people diagnosed with schizophrenia, recruited in the real-world multicenter study of the Italian Network for Research on Psychoses. In particular, the study compared subjects with low, moderate, and prominent ASD symptoms, as defined by the PAUSS cut-off scores for nonautistic and autistic schizophrenia, on demographic and clinical variables and on neurocognitive, sociocognitive, and real-world functional measures.

## Methods

### Sample

For this study, the database of the Italian Network for Research in Psychoses was used. It includes 921 individuals diagnosed with schizophrenia (280 females, mean age 40.17 ± 10.71).

The Italian Network for Research in Psychoses is a large research network including 26 Italian University Psychiatric Clinics and Mental Health Departments, providing data on a large number of people diagnosed with schizophrenia living in the community that has been assessed with a wide array of clinical, cognitive, and functional measures [27,28].

Participants were recruited from March 1, 2012 to September 30, 2013.

Inclusion criteria were: (a) diagnosis of schizophrenia according to DSM-IV TR criteria [29] confirmed with the structured clinical interview for DSM-IV-patient version (SCID-I-P) [30] and (b) age between 18 and 66 years. Exclusion criteria were: (a) history of head trauma with loss of consciousness; (b) history of moderate to severe mental retardation or of neurological diseases; (c) history of alcohol and/or substance abuse in the last 6 months; (d) current pregnancy or lactation; (e) inability to provide informed consent for participation in the study; (f) treatment modifications and/or hospitalization due to symptom exacerbation in the last 3 months.

According to the same procedure in all centers, enrolled patients completed the assessments in 3 days with the following schedule: collection of sociodemographic information, psychopathological evaluation, and neurological assessment on day 1, in the morning; assessment of neurocognitive functions, social cognition, and functional capacity on day 2, in the morning; assessment of personal resources and perceived stigma either on day 3 (morning or afternoon) or in the afternoon of day 1 or 2, according to the patient’s preference. For real-life functioning assessment, patient’s key caregiver was invited to join one of the scheduled sessions.

Out of 1,691 screened patients, 1,180 were eligible; of these, 202 refused to participate, 57 dropped out before completing the procedures, and 921 were included in the analyses.

All included subjects provided written informed consent to participate after receiving a comprehensive explanation of study procedures and goals. The study protocol was approved by the Ethical Committee of the coordinating center and of the other participating centers (approval number 73/2012).

Demographic and clinical characteristics of the sample are reported in Table 1.

**Table 1.** Characteristics of the sample (*N* = 921).

Variable	%, Mean ± SD
Gender (% females)	30.40
Age (years, mean ± SD)	40.17 ± 10.71
Education (years, mean ± SD)	11.61 ± 3.43
Work (% employed)	29.23
Age at first psychotic episode (years, mean ± SD)	24.02 ± 7.19
Previous hospitalization (% yes)	68.39
Number of previous hospitalizations (Mean ± SD)	3.77 ± 4.33
Complete remission at first episode (% yes)	38.26
Suicide attempt, lifetime (% yes)	17.12
Previous alcohol abuse (% yes)	16.41
Previous substance abuse (% yes)	25.95

### Measures

#### Clinical assessment

Demographic and clinical data for each subject were collected from different sources, such as family members, medical records, and mental health worker reports.

The PANSS [25] was used for the assessment of symptoms severity. The PANSS is a semistructured interview composed by 30 items divided in three subscales, namely positive symptoms, negative symptoms, and general psychopathology. Each item is accompanied by a specific definition and by detailed anchoring criteria for each rating point, ranging from “absent” (1) to “severe” (7).

#### ASD symptoms assessment

In order to assess the severity of ASD symptoms, the PANSS Autism Severity Scale (PAUSS) [24] was derived from the PANSS and calculated by performing the sum of the following PANSS items: N1 (“blunted affect”), N3 (“poor rapport”), N4 (“social withdrawal”), N5 (“difficulties in abstract thinking”), N6 (“lack of spontaneity and flow of conversation”), N7 (“stereotyped thinking”), G5 (“mannerism”), and G15 (“preoccupation”). The PAUSS validity in identifying ASD symptoms in people diagnosed with schizophrenia has been already demonstrated and found to be satisfying, with the PAUSS strongly correlating with other more established diagnostic tools for the assessment of ASD and showing even better sensitivity than such scales in detecting ASD symptoms in people with schizophrenia [26].

According to the results of the original validation study of the PAUSS [24], subjects were divided into three different groups, based on the PAUSS total score: subjects with “autistic schizophrenia” (PAUSS ≥30), subjects with “nonautistic schizophrenia” (PAUSS ≤10), and subjects with “moderate ASD symptoms” (PAUSS between 11 and 29). These cut-off scores have been identified and validated by the scale authors in a large sample of individuals diagnosed with schizophrenia and have been reported in the scale validation study [24].

#### Cognitive assessment

Cognitive performance was assessed using the MATRICS consensus cognitive battery (MCCB) [31]. The MCCB is composed by specific tasks assessing the following cognitive domains: speed of processing (Trail Making Test Part A; Brief Assessment of Cognition in Schizophrenia: Symbol Coding; Category Fluency Test: Animal Naming), verbal and spatial learning (Hopkins Verbal Learning Test-Revised, immediate recall; Brief Visuospatial Memory Test-Revised), reasoning and problem solving (Neuropsychological Assessment Battery, Mazes subtest), attention (Continuous Performance Test: Identical Pairs), working memory (Wechsler Memory Scale, Spatial Span subset; Letter Number Span Test), and social cognition (Mayer–Salovey–Caruso Emotional Intelligence Test: Managing Emotion task). A *t*-score was computed for each cognitive domain, corrected by gender, age, and education, and a global cognitive composite score was finally calculated following the recommendation of the battery developers [32].

#### Functional outcomes measures

Functional capacity was assessed with the UCSD performance-based skills assessment, brief (UPSA-B) [33]. The UPSA-B is a brief and widely used performance-based instrument that assesses skills involved in community tasks: “financial skills” (e.g., counting money and paying bills) and “communication skills” (e.g., to dial a telephone number for emergency or reschedule an appointment by telephone), with a total score ranging from 0 to 100.

Real-world functioning was assessed using the Specific Level of Functioning Scale (SLOF), an informant-rated measure that explores different aspects of functioning and is based on the key caregiver’s judgment on behavior and functioning of patients [34]. It consists of 43 items, divided into six different scales, including the following domains: physical efficiency, skills in self-care, interpersonal relationships, social acceptability, participation in community activities (e.g., shopping, using public transportation), and working abilities. Each item is rated from 1 to 5, with higher scores indicating better functioning. The SLOF has been found to be a reliable and valid instrument to assess real-world functioning in people diagnosed with schizophrenia, with good construct validity and internal consistency, and has been recently validated in Italian [35].

### Data collection and handling

Comparability of data collection procedures was assured by a centralized training of all the researchers, before starting recruitment and assessments. For each category of variables (psychopathology, including diagnosis, illness-related factors, cognition, real-life functioning, personal resources, and context-related factors), at least one researcher per site was trained. In order to avoid halo effects, the same researcher could not be trained for more than one category. The interrater reliability was formally evaluated by Cohen’s kappa for categorical variables and intraclass correlation coefficient (ICC) or percentage agreement for continuous variables. An excellent interrater agreement was found for the SCID-I-P (Cohen’s kappa 0.98). Good to excellent agreement among raters was observed for SLOF (ICC 0.55–0.99, percentage agreement 70.1–100%); PANSS (ICC 0.61–0.96, percentage agreement 67.7–93.5%); and MCCB (ICC 0.87).

Assessment was conducted within 2 weeks after subjects’ recruitment.

In the database of the study, fields for all variables were exactly corresponding to those of paper forms on which data were collected. For variables with finite domain and low cardinality, raw data were inputted to the database by means of drop-down menu showing the possible relevant options, while for a minority of variables, with finite domain and high cardinality or with bounded domain, digits were typed in the database. In both cases, the system verified that the input was admissible against extreme values. For cases in which admissibility was not verifiable (e.g., a variable for which high values are still possible although improbable), the quality control periodically performed for all variables allowed to identify outlier values that were then checked against data on paper.

Data of subjects were associated to a pseudonym (ID code), and the correspondence between ID code and the subject was on an off-system paper form located at the site that recruited the subject. Data in transit between the remote location and the server were encrypted with the end-to-end coding. A full backup of the database was performed everyday and signed off.

All participants signing the informed consent to participate in the study gave their authorization to publication of results in scientific journals. No deadline for data analysis or publication was specified in the informed consent.

### Statistical analyses

The three groups of subjects identified using the PAUSS cut-off scores were compared on demographic, clinical, cognitive, and functional measures. The distribution of scores of each considered variable was inspected for normality and for homogeneity of variance in order to allow the use of parametric statistics.

Dichotomous variables were analyzed using Pearson’s *χ* ^2^ tests, with results reported as percentages. Continuous variables were analyzed with general linear model analyses of co-variance (ANCOVAs). A construct calculated by subtracting the PAUSS total score from the PANSS total score (PANSS minus PAUSS) was included as a covariate in the analyses of cognitive performance and functioning, in order to rule out the possibility that the PAUSS could represent and indirect proxy of global symptoms severity. This construct was introduced in the analyses instead of the total PANSS score in order to avoid collinearity with the PAUSS, as detailed in a previous study on the role of ASD symptoms in people with schizophrenia [18]. Age and education were also included as covariates in the analyses on functioning but not in the analyses regarding cognitive performance, since cognitive performance variables were already corrected by gender, age, and education. Posthoc, between-groups analyses were performed accounting for multiple comparisons using Bonferroni correction.

Statistical analyses were performed using SPSS 15.0. *p*-Values <0.05 (two tailed) were considered significant.

## Results

### Prevalence of ASD symptoms

The mean PAUSS total score was 22.89 (SD ± 8.26). One hundred and eighty-five subjects (20.11% of the total sample) had a PAUSS ≥30 and thus were included in the “autistic schizophrenia” group; 56 subjects (6.09%) had a PAUSS ≤10 and thus were included in the “nonautistic schizophrenia” group; 679 (73.80%) had a PAUSS between 11 and 29 and thus were included into the “moderate ASD symptoms” group.

### Between-groups comparisons of demographic variables

For demographic variables (Table 2), significant between-groups differences emerged for age (*p* < 0.001), with “nonautistic schizophrenia” subjects being younger than “autistic schizophrenia” (*p* < 0.001) and then “moderate ASD symptoms” patients (*p* = 0.003), education (*p* = 0.001), with “autistic schizophrenia” subjects showing fewer years of education compared to “moderate ASD symptoms” (*p* = 0.010) and to “nonautistic schizophrenia” subjects (*p* = 0.004), and employment (*p* = 0.004), with a larger proportion of unemployed subjects in the “autistic schizophrenia” group, compared to “moderate ASD symptoms” (*p* = 0.009) and to “nonautistic schizophrenia” (*p* = 0.009) groups. No differences emerged for gender distribution among groups.

**Table 2.** Group comparison for demographic and clinical variables.

	Non-Aut-S	Moderate	Aut-S	ANOVA/Pearson *χ* ^2^	Non-Aut-S vs moderate	Moderate vs Aut-S	Non-Aut- S vs Aut-S
Variable	Mean ± SD/ %(*n*)	Mean ± SD/ %(*n*)	Mean ± SD/ %(*n*)	(*p*-value)	(*p*-value)	(*p*-value)	(*p*-value)
*Gender*							
Male	62.50 (35)	69.26 (471)	72.97 (135)	0.307	0.891	0.969	0.396
Female	37.50 (21)	30.74 (209)	27.03 (50)				
*Age* (years)	35.30 ± 1.43	40.16 ± 0.41	41.46 ± 0.78	<0.001**	0.003**	0.265	<0.001**
*Education* (years)	12.55 ± 3.06	11.73 ± 3.46	10.90 ± 3.27	0.001**	0.249	0.010*	0.004**
*Work*							
Employed	39.29 (22)	30.96 (203)	19.89 (36)	0.004**	0.600	0.009**	0.009**
Unemployed	60.71 (34)	69.04 (453)	80.11 (145)				
*Age at first psychotic episode* (years)	25.08 ± 0.96	24.14 ± 0.27	23.20 ± 0.53	0.149	1.000	0.346	0.260
*Previous hospitalization*							
Yes	51.78 (29)	69.59 (467)	69.06 (125)	0.022*	0.018*	1.000	0.054
No	48.22 (27)	30.41 (204)	30.94 (56)				
*Previous hospitalizations* (number)	2.75 ± 3.63	3.85 ± 4.40	3.75 ± 4.21	0.381	0.497	1.000	0.726
*Complete remission at first episode*							
Yes	65.45 (36)	38.66 (254)	27.71 (46)	<0.001**	<0.001**	0.024*	<0.001**
No	34.55 (19)	61.34 (403)	72.29 (120)				
*Suicide attempt, lifetime*							
Yes	16.36 (9)	17.95 (121)	14.28 (26)	0.501	1.000	0.723	1.000
No	83.44 (46)	82.05 (553)	85.72 (156)				
*Previous alcohol abuse*							
Yes	19.64 (11)	17.01 (115)	13.18 (24)	0.371	1.000	0.684	0.699
No	80.36 (45)	82.99 (561)	86.82 (158)				
*Previous substance abuse*							
Yes	28.57 (16)	27.21 (185)	20.54 (38)	0.167	1.000	0.222	0.810
No	71.43 (40)	72.79 (495)	79.46 (147)				

Posthoc comparisons include Bonferroni correction.

Abbreviations: Aut-S, autistic schizophrenia; Moderate, moderate ASD symptoms; Non-Aut-S, nonautistic schizophrenia.

* *p* < 0.05

** *p* < 0.01.

### Between groups comparisons of clinical variables

For clinical variables (Table 2), significant between-groups differences emerged in the rate of individuals having previous hospitalizations (*p* = 0.022), which were lower in the group of “nonautistic schizophrenia” compared with the “moderate ASD symptoms” subjects (*p* = 0.018). A complete remission at first episode was also different between-groups (*p* < 0.001) and was achieved more frequently in “nonautistic schizophrenia” subjects, compared to the “autistic schizophrenia” (*p* < 0.001) and to the “moderate ASD symptoms” group (*p* < 0.001); the latter showing still significantly higher remission rate than that of the “autistic schizophrenia” group (*p* = 0.024). No between-groups differences emerged for age at first psychotic episode, number of previous hospitalizations, previous suicide attempts, and previous alcohol and substance abuse.

### Between groups comparisons of cognitive performance

Between-groups comparisons of cognitive measures (Table 3) were covaried by nonautistic symptoms severity (PANSS minus PAUSS). Significant between-groups differences at the ANCOVAs were observed on different cognitive domains, in particular on processing speed (*p* = 0.010), attention (*p* = 0.011), verbal memory (*p* = 0.035), and social cognition (*p* = 0.001). A significant between-group difference was also observed on the global cognition composite score (*p* = 0.010).

**Table 3.** Group comparison for cognitive measures.

	Non-Aut-S	Moderate	Aut-S	ANCOVA	Non-Aut-S vs moderate	Moderate vs Aut-S	Non-Aut-S vs Aut-S
Variable	Mean ± SD	Mean ± SD	Mean ± SD	(*p*-value)	(*p*-value)	(*p*-value)	(*p*-value)
*Processing speed* (*t*-score)	35.00 ± 10.91	31.95 ± 11.07	26.96 ± 12.20	0.010*	1.000	0.010*	0.071
*Attention* (*t*-score)	40.70 ± 13.90	37.67 ± 11.04	33.06 ± 10.76	0.011*	0.975	0.012*	0.057
*Working memory* (*t*-score)	38.61 ± 12.01	35.62 ± 11.45	30.55 ± 12.73	0.087	1.000	0.083	0.442
*Verbal memory* (*t*-score)	39.70 ± 11.42	35.44 ± 11.74	31.94 ± 12.88	0.035*	0.234	0.140	0.037*
*Visual memory* (*t*-score)	35.54 ± 14.78	32.83 ± 14.42	28.69 ± 15.04	0.590	1.000	0.914	1.000
*Problem solving* (*t*-score)	39.91 ± 9.29	38.26 ± 10.25	34.95 ± 9.18	0.088	1.000	0.086	0.409
*Social cognition* (*t*-score)	36.69 ± 7.17	32.47 ± 6.98	31.50 ± 7.36	0.001**	0.001**	1.000	0.002**
*Global cognition* (composite score)	31.47 ± 13.16	26.93 ± 11.43	21.46 ± 12.31	0.010*	0.517	0.017*	0.028*

Raw scores for each variable are reported; all cognitive measures are corrected for gender, age, education; all the analyses were covaried by nonautistic symptoms severity (PANSS–PAUSS). Post-hoc comparisons include Bonferroni correction.

Abbreviations: Aut-S, autistic schizophrenia; Moderate, moderate ASD symptoms; Non-Aut-S, nonautistic schizophrenia.

* *p* < 0.05.

** *p* < 0.01.

When performing posthoc comparisons, a poorer cognitive performance in the “autistic schizophrenia” group, compared to “nonautistic schizophrenia” subjects emerged for verbal memory (*p* = 0.037), social cognition (*p* = 0.002), and global cognition (*p* = 0.028). A poorer cognitive performance in the “autistic schizophrenia” group, compared to subjects with “moderate ASD symptoms,” emerged for processing speed (*p* = 0.010), attention (*p* = 0.012), and global cognition (*p* = 0.017). A poorer cognitive performance in subjects with “moderate ASD symptoms” compared with “nonautistic schizophrenia” subjects emerged in the social cognition domain (*p* = 0.001).

No between-groups differences emerged for working memory, visual memory, and problem solving.

### Between groups comparisons of psychosocial functioning

Between-groups comparisons of psychosocial functioning measures (Table 4) were covaried by age, education, and nonautistic symptoms severity (PANSS minus PAUSS).

**Table 4.** Group comparison for functional measures.

	Non-Aut-S	Moderate	Aut-S	ANCOVA	Non-Aut-S vs moderate	Moderate vs Aut-S	Non-Aut-S vs Aut-S
Variable	Mean ± SD	Mean ± SD	Mean ± SD	(*p*-value)	(*p*-value)	(*p*-value)	(*p*-value)
*UPSA-B* (functional capacity)	80.12 ± 14.77	69.52 ± 21.22	57.49 ± 22.22	0.004*	0.577	0.006**	0.021*
*SLOF: Physical functioning* (real-world physical efficiency)	24.61 ± 0.80	24.28 ± 1.23	23.99 ± 1.77	0.938	1.000	1.000	1.000
*SLOF: Personal care* (real-world self-care skills)	33.86 ± 2.19	32.08 ± 3.56	29.58 ± 5.20	0.056	1.000	0.049	0.517
*SLOF: Interpersonal relationships* (real-world interpersonal skills)	27.60 ± 6.24	22.83 ± 5.59	18.85 ± 5.93	< 0.001**	< 0.001**	< 0.001**	< 0.001**
*SLOF: Social acceptability* (real-world social acceptability)	33.16 ± 3.06	32.57 ± 3.21	31.86 ± 3.45	0.019*	0.312	0.050	0.028*
*SLOF: Activities* (participation in community activities)	51.45 ± 5.00	46.93 ± 7.48	40.48 ± 10.38	<0.001*	1.000	<0.001**	0.013*
*SLOF: Work* (real-world working skills)	23.98 ± 5.59	20.39 ± 6.05	17.33 ± 5.87	0.776	1.000	1.000	1.000

Raw scores for each variable are reported; all the analyses were covaried by age, education, and nonautistic symptoms severity (PANSS–PAUSS). Posthoc comparisons include Bonferroni correction.

Abbreviations: Aut-S, autistic schizophrenia; Moderate, moderate ASD symptoms; Non-Aut-S, nonautistic schizophrenia; SLOF, Specific Level of Functioning Scale; UPSA-B, UCSD performance-based skills assessment-brief version.

* *p* < 0.05.

** *p* < 0.01.

Significant between-groups differences were observed on functional capacity, as measured by the UPSA-B (*p* = 0.004), on real-world interpersonal skills, as measured by the SLOF-interpersonal relationships subscale (*p* < 0.001), on social acceptability, as measured by the SLOF-social acceptability subscale (*p* = 0.019), and on participation in community activities, as measured by the SLOF-activities subscale (*p* < 0.001).

Looking at the posthoc comparisons, a poorer psychosocial performance of the “autistic schizophrenia” group compared to “nonautistic schizophrenia” subjects emerged in different areas, as measured with the UPSA-B (*p* = 0.021), the SLOF-interpersonal relationships (*p* < 0.001) and the SLOF-activities (*p* = 0.013). Also, a poorer psychosocial performance of the “autistic schizophrenia” group compared to the “moderate ASD symptoms” group emerged for the UPSA-B (*p* = 0.006), the SLOF-interpersonal relationships (*p* < 0.001), and the SLOF-activities (*p* < 0.001). Finally, “moderate ASD symptoms” subjects showed a poorer psychosocial performance than “nonautistic schizophrenia” subjects in the SLOF-interpersonal relationships (*p* < 0.001). A better psychosocial performance of the “autistic schizophrenia” group compared to the “nonautistic schizophrenia” group emerged for the SLOF-social acceptability (*p* = 0.028). No between-groups differences emerged in the SLOF-physical functioning, the SLOF-personal care, and the SLOF-work.

## Discussion

The study demonstrated that the cut-off scores of the PAUSS allowed to identify, among a large and representative sample of subjects with schizophrenia, three groups of patients with different clinical, cognitive, and functional characteristics.

The between-groups differences emerged in the neuro- and social-cognitive measures, as a whole, corroborated the hypothesis of greater cognitive impairment in people diagnosed with schizophrenia and increasingly prominent ASD symptoms [20,26]. They are in line with previous findings correlating ASD features and deficits in neuro- and social-cognition [36–38] and suggest a direct relationship between impairment in social cognitive performance and ASD symptoms severity in people diagnosed with schizophrenia [18].

As for functional outcomes, functional capacity and personal and social functioning impairments were found to progressively increase with the severity of ASD symptoms. These results confirm those of previous studies conducted in much smaller samples [20,26], that hypothesized the existence of a gradient of increasingly higher impairment in people with schizophrenia with greater levels of ASD symptoms severity.

Considering real-world functioning, the greater impairment in interpersonal relationships and participation in community activities in individuals with more severe ASD symptoms was an expected result, as deficits in social interactions are one of the key features of ASD and is in line with previous findings [19].

The use of age, education, and nonautistic symptoms severity as covariates in the analyses allowed subjects with more prominent ASD features to emerge as the group showing better social acceptability. This result might at first appear counterintuitive also considering the raw scores obtained with the scale. In fact, the between-groups differences, even when corrected by age, education, and nonautistic symptoms were small in size and clinically negligible and probably sensitive to the statistic procedure applied. Anyway, it may be well that the usual social interaction style of ASD individuals, more prone to social retirement, could be perceived to some extent as more socially acceptable than that of other groups of people diagnosed with schizophrenia.

No difference between PAUSS subgroups was observed in the SLOF-work subscale; however, individuals with more prominent ASD symptoms where more frequently unemployed. This may reflect both the overall low level of working skills of the entire sample, as indicated by the SLOF-work subscale, and the fact that other factors, not directly related to work skills, may interfere with the possibility to maintain a job in subjects with prominent ASD symptoms. This is an intriguing perspective that should be better investigated with specific studies.

In general, our results showed a high prevalence of ASD symptoms in people diagnosed with schizophrenia, confirming the existence of significant areas of overlap between SSDs and ASDs. They also confirmed the possibility to use the PAUSS, a simple, fast, and practical tool for the assessment of ASD symptoms in people diagnosed with schizophrenia, for identifying subgroups of subjects diagnosed with schizophrenia with increasing ASD symptoms severity and a parallel gradient of severity of cognitive and psychosocial functioning impairments. These results corroborate, in a clinical perspective, those of studies focused on neurobiological aspects of ASD features in schizophrenia, as the PAUSS has been recently used in genetic studies for the investigation of the association between the autistic genotype and phenotype [39,40], and in neuroimaging studies, which reported an association between autistic symptoms and structural and functional imaging features [41,42].

To our knowledge, this is the first study in which a gradient of increasingly higher cognitive and functional impairment among different levels of ASD symptoms severity has been demonstrated in people diagnosed with schizophrenia.

Among the strengths of the study are the characteristics of the sample analyzed, composed of a very large group of well-diagnosed subjects diagnosed with schizophrenia, representative of the heterogeneous demographic and clinical characteristics of the Italian population of people diagnosed with schizophrenia, in the real-world. Moreover, the cognitive and functioning assessment was conducted using a wide array of well-validated instruments, allowing a reliable investigation of specific cognitive and functioning areas.

It is possible that the correlation between ASD symptoms severity and cognitive and functional deficits might be partially explained by a longer duration of illness, as individuals in the “nonautistic schizophrenia” group were younger, and possibly in an earlier stage of the disorder: therefore, they could have not yet developed the negative cognitive and functional sequelae, as well as a more severe clinical condition, which are usually associated with longer term psychosis. However, by introducing covariates such as age, education, and nonautistic symptoms severity in the analyses, we were able to rule out the influence of one or more of such covariates, thus increasing the specificity of the results.

The study has also some limitations. First, it was not specifically designed to test the validity of the PAUSS nor to apply it as a measure of ASD symptoms. Second, some domains of social cognition that are typically impaired in people diagnosed with schizophrenia, such as attributional style bias and social perception were not included in the assessment of social cognition. Third, given the cross-sectional design of the present study, no prospective observation was performed; therefore, no longitudinal evaluation of the course and trajectory of ASD symptoms in people diagnosed with schizophrenia could be made. This did not allow to further contribute to the debate regarding the nature of ASD symptoms in schizophrenia as a state or trait variable. Finally, no evaluation of the effect of treatment on ASD symptoms was performed.

Beyond these limitations, the results of this study strengthen the notion of the relevant impact of ASD symptoms on different aspects of the disease, crucial to the life of people diagnosed with schizophrenia, and suggest that prominent ASD symptoms could characterize a subpopulation of individuals with SSD.

Future studies should focus on observing the course of ASD symptoms in people diagnosed with schizophrenia in a longitudinal perspective, on evaluating the effects of different treatments on ASD symptoms and on assessing the presence of ASD symptoms in relatives of people diagnosed with schizophrenia in order to estimate the familial component of a possible autistic phenotype of schizophrenia. Even more important from a clinical point of view could be to analyze whether individuals identified on the basis of different severity of PAUSS could have different response to pharmacologic treatment or to specific psychosocial interventions.

## Data Availability

Data that support the findings of this study are not available.

## Appendix

Members of the Italian Network for Research on Psychoses involved in this study include: Anna Ceraso, Alessandro Galluzzo, Jacopo Lisoni (University of Brescia); Piergiuseppe Di Palo, Marco Papalino, Raffaella Romano (University of Bari); Federica Pinna, Alice Lai, Silvia Lostia di Santa Sofia (University of Cagliari); Paola Bucci, Giuseppe Piegari, Francesco Brando, Luigi Giuliani (University of Campania “L. Vanvitelli,” Naples); Maria Salvina Signorelli, Laura Fusar Poli (University of Catania); Giovanni Martinotti, Mauro Pettorruso, Chiara Montemitro (University of Chieti); Mario Altamura, Stefania Malerba, Flavia Padalino (University of Foggia); Andrea Amerio, Pietro Calcagno, Domenico Zampogna (University of Genoa); Laura Giusti, Anna Salza, Silvia Mammarella, Francesca Pacitti, Valentina Socci, Dalila Talevi (University of L’Aquila); Carla Gramaglia, Alessandro Feggi, Amalia Jona (University of Eastern Piedmont, Novara); Angela Favaro, Elena Tenconi, Paolo Meneguzzo (University of Padua); Paolo Ossola, Matteo Tonna, Maria Lidia Gerra (University of Parma); Claudia Carmassi, Camilla Gesi, Barbara Carpita (University of Pisa); Giulio Corrivetti, Giammarco Cascino, Gianfranco del Buono (Department of Mental Health, Salerno); Fabio Di Fabio, Antonio Buzzanca, Nicoletta Girardi, Roberto Brugnoli, Anna Comparelli, Valentina Corigliano (Sapienza University of Rome); Andrea Fagiolini, Simone Bolognesi, Arianna Goracci (University of Siena); Giorgio Di Lorenzo, Cinzia Niolu, Michele Ribolsi (Tor Vergata University of Rome); Claudio Brasso, Cecilia Riccardi, Elisa Del Favero (University of Turin).
